# Research and Remedies

**Published:** 1912-06-22

**Authors:** 


### RESEARCH AND REMEDIES.

### Lead Poisoning from Snuff.

A curious case of fatal lead poisoning has been reported by Stadler, and is abstracted in the *Medical Review*. A woman, aged thirty-three, was found to be suffering from diplopia, which began suddenly three weeks before with headache, vomiting, vertigo, and a feeling of anxiety. Nearly two years before she had had an attack of colic with constipation and anæmia. She was suffering from paresis of both sixth nerves, and from bilateral optic neuritis with retinal hæmorrhages. There was nothing abnormal in the urine; but there was a partial bluish discolouration of the gums. A diagnosis of plumbism was made, but the source of the poisoning could not be traced: the husband and five children were quite free from symptoms. She reacted well to treatment, and in ten weeks was quite well; even the optic neuritis disappeared. Two months after the latter date she had an abortion; and in another month she became somewhat suddenly unconscious. After a slight temporary improvement she relapsed and died in a week. It was then discovered that the patient had been in the habit of snuffing inordinately. She bought her snuff in tinfoil packets, which she was accustomed to put in her pocket; she would then abstract a pinch frequently direct from the pocket. Examination of a half-used packet showed that it contained loose fragments of tinfoil; and, in addition, it was proved that the moist alkaline snuff had corroded the metal foil, and actually contained nearly 2 per cent. of lead. Calculation showed that her average daily dose of snuff would contain about seventeen times enough lead to produce plumbism; so that, in spite of wastage, it was evident that the snuff was the cause of the symptoms. No other case of lead poisoning could be traced among devotees of this particular brand of snuff.

### The Antagonism Between Mammary and Ovarian Function.

Under this title the *Medical Review* gives a *résumé* of a remarkable case, reported from Germany by Vogt, which illustrates the obscure and at present poorly understood relations between the mammary glands and the ovaries. The cessation of menstruation during pregnancy and lactation is, of course, quite familiar; and this is believed to be due to ovarian influence, not to the mere mechanical interference with the endometrium which pregnancy entails; the amenorrhœa of ectopic gestation is evidence of this. The case in question is that of a woman whose third child was four and a half months old when she sustained severe injuries to the whole of one breast owing to the explosion of a tin of spirit. She had ceased to nurse her child at six weeks of age, and had menstruated regularly thereafter; she had not become again pregnant. The burn was extensive and of the third degree. Immediately after the accident both breasts, then quiescent, began to secrete profusely, and menstruation stopped. The burns healed very slowly, and were not completely cicatrised for fourteen months. Galactorrhœa and amenorrhœa persisted throughout this interval. As soon as the wounds were completely covered with epithelium the secretion of milk ceased; two days later menstruation began and continued normally thenceforward. A subsequent pregnancy began about a year and a half after that time. The curious interdependence, or rather antagonism, between lactation and ovulation is particularly well shown by this case, because it was not complicated by either pregnancy or the puerperium. Another illustration of the intimate connection between the mammary and genital function is provided by the well-known fact that in some women each application of the baby to the nipple (during the early days of the puerperium) provokes an after-pain in the uterus.

### The Rumpell-Leede Sign of Scarlet Fever.

Some three years ago Rumpell drew attention to the fact that hæmorrhages will occur into the skin of a scarlet-fever patient if a Bier's stasis bandage be applied over the elbow joint. This test was recommended as of diagnostic value in doubtful cases. The matter was further investigated by Leede, who concluded that a negative result is proof positive against scarlet fever, but that a positive result is not in itself conclusive. Several other authors have published similar experiences. Dr. May Michael, writing in *Archives of Pediatrics*, finds, after experimenting on a hundred normal children, that hæmorrhages on the anterior surface of the elbow can be produced in practically all normal children by applying sufficient pressure; also that the amount of pressure advised by Leede produces hæmorrhages in 60 per cent. of normal children. A recent research by Strauch confirms these figures; so it is evident that Rumpell's test is one which is not likely to prove of any material service in the diagnosis of scarlet fever.

### Postural Treatment of Transverse Presentations.

For some time past Dr. A. F. A. King, of Washington, has been an advocate of a special postural treatment of the transverse presentation in obstetric work. Internal version is, as every accoucheur knows, always slightly risky; so that any method which involves no internal manipulations is worth trying first of all. Where the head rests on the right iliac fossa, Dr. King makes the patient place her left foot flat upon the ground and somewhat in advance of the body. She then squats on the right heel by resting it on the toes only, and just under the buttock. The pressure of the thighs upon the abdomen is said to result in correction of the malpresentation. Especially the author pleads for the instruction of midwives and nurses in this postural treatment, so that it could be given a trial while waiting for the advent of the obstetrician. The full account of the plan appears in the *Interstate Medical Journal*, and it seems that the author has advocated it in several other American periodicals.

